# Glutathione and peroxisome redox homeostasis

**DOI:** 10.1016/j.redox.2023.102917

**Published:** 2023-10-04

**Authors:** Maria J. Ferreira, Tony A. Rodrigues, Ana G. Pedrosa, Ana R. Silva, Beatriz G. Vilarinho, Tânia Francisco, Jorge E. Azevedo

**Affiliations:** a Instituto de Investigação e Inovação em Saúde (I3S), Universidade do Porto, Rua Alfredo Allen, 208, 4200-135, Porto, Portugal; b Instituto de Biologia Molecular e Celular (IBMC), Universidade do Porto, Rua Alfredo Allen, 208, 4200-135, Porto, Portugal; c Instituto de Ciências Biomédicas de Abel Salazar (ICBAS), Universidade do Porto, Rua de Jorge Viterbo Ferreira, 228, 4050-313, Porto, Portugal

**Keywords:** Glutathione, Peroxisomes, Redox homeostasis, Membrane permeability, Antioxidant

## Abstract

Despite intensive research on peroxisome biochemistry, the role of glutathione in peroxisomal redox homeostasis has remained a matter of speculation for many years, and only recently has this issue started to be experimentally addressed. Here, we summarize and compare data from several organisms on the peroxisome-glutathione topic. It is clear from this comparison that the repertoire of glutathione-utilizing enzymes in peroxisomes of different organisms varies widely. In addition, the available data suggest that the kinetic connectivity between the cytosolic and peroxisomal pools of glutathione may also be different in different organisms, with some possessing a peroxisomal membrane that is promptly permeable to glutathione whereas in others this may not be the case. However, regardless of the differences, the picture that emerges from all these data is that glutathione is a crucial component of the antioxidative system that operates inside peroxisomes in all organisms.

## Highlights

• The protein repertoire of GSH-dependent peroxisomal enzymes in different organisms varies widely.
• Different organisms may use different strategies to maintain a reduced pool of GSH inside peroxisomes.
• Despite all the differences, GSH is a crucial component of the peroxisomal antioxidant system in all organisms.

## Graphical abstract

**Figure:**
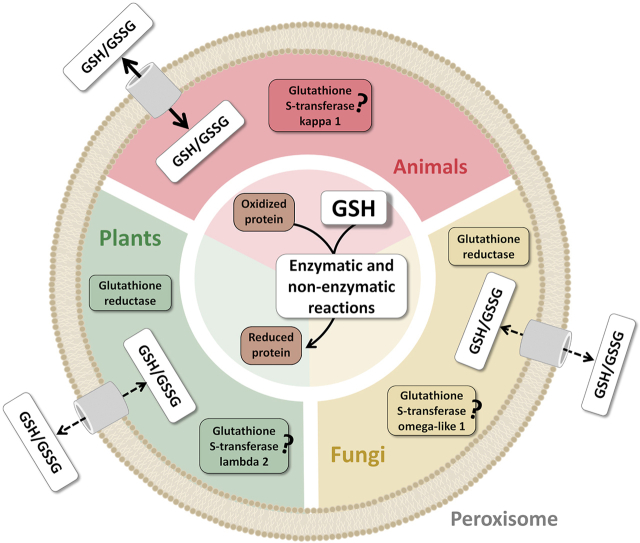

## 1 Introduction

**Fig. 1:**
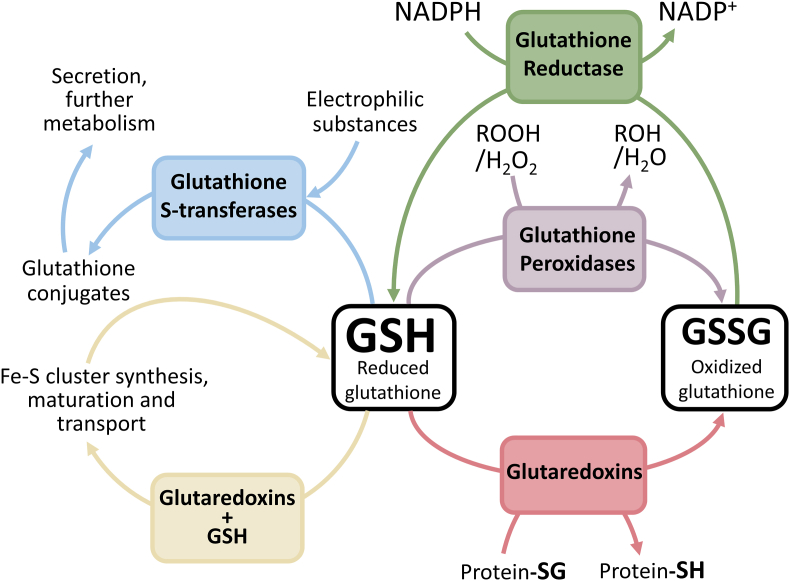
**– The functions of glutathione.** Glutathione, the most abundant thiol in most organisms, plays a role in multiple pathways/processes such as iron-sulfur (Fe–S) cluster biogenesis and transport (yellow), detoxification of electrophilic substances (blue) and hydroperoxides (purple) and protein deglutathionylation (pink). In many of these reactions, reduced glutathione (GSH) is oxidized to GSSG, which must then be reduced back to GSH by the NADPH-dependent glutathione reductase (green). ROOH and ROH, organic hydroperoxides and the corresponding alcohols, respectively; Protein-SG and Protein-SH, glutathionylated and reduced protein, respectively. (For interpretation of the references to color in this figure legend, the reader is referred to the Web version of this article.)

Glutathione is the most abundant low-molecular weight thiol-containing metabolite in the majority of organisms [1,2]. Besides participating in the biogenesis of iron-sulfur proteins and in the detoxification of a myriad of electrophilic metabolites and drugs, glutathione is also a key antioxidant molecule used by a variety of enzymes to neutralize reactive oxygen species (ROS) and to control the redox state of cysteine residues in proteins [2,3]. In many of these reactions, the reduced form of glutathione (GSH) is oxidized to glutathione disulfide (GSSG) which is then subsequently converted to GSH by a NADPH-dependent glutathione reductase ([3,4]; see Fig. 1).

Glutathione is exclusively synthesized in the cytosol of non-plant eukaryotic cells where its concentration reaches millimolar levels [[5], [6], [7], [8]]. Despite its cytosolic origin, glutathione is also found in the lumen of several subcellular organelles. The mechanisms that allow organelles to acquire cytosolic glutathione are still unclear [7,9]. Nevertheless, it is known that in some cases GSH and/or GSSG do not diffuse freely across the corresponding membranes, thus leading to situations in which the cytosolic and organelle pools of glutathione are not in redox equilibrium. One of these cases is the endoplasmic reticulum (ER), the membrane of which is permeable to GSH but less so to GSSG [[10], [11], [12]]. Thus, any GSSG produced in the ER lumen (*e.g.*, in ROS-scavenging and thiol-disulfide exchange reactions; [12,13]) is not promptly exported to the cytosol where it would be reduced by cytosolic glutathione reductase [11,13]. Ultimately, this leads to an increase of the GSSG/GSH ratio in the ER lumen thus reflecting and/or controlling the oxidative environment that is required for disulfide bond formation in proteins that follow the secretory pathway (reviewed in Ref. [14]). Another case regards the mitochondrial matrix. Indeed, the mitochondrial inner membrane displays a very restricted permeability to GSH/GSSG, and thus the mitochondrial matrix pools of GSH and GSSG are essentially kinetically independent of the cytosolic ones [7,15,16]. In this case, however, there are no obstacles to the reduction of GSSG because there is a NADPH-dependent glutathione reductase in the mitochondrial matrix [3].

The situation regarding peroxisomes is now starting to be explored. It was long known that glutathione plays some role in the lumen of these organelles in all organisms characterized up to now, including yeasts, plants, and mammals. However, the question of whether the peroxisomal pool of glutathione is kinetically insulated from the cytosolic one remained unexplored until recently. Strikingly, there is no single answer for this question. Indeed, the available data suggest that in some organisms the peroxisomal and glutathione pools may display poor kinetic connectivity, whereas in others this seems not to be the case. Knowledge on this and other aspects of glutathione biology (*e.g.*, the glutathione-dependent enzyme repertoire inside peroxisomes) is of crucial importance for our understanding of how peroxisomes maintain redox homeostasis and keep their cysteine-containing proteins in an active state. Here, we discuss recent findings on this topic giving an emphasis to the mammalian peroxisome.

## 2 The mammalian peroxisomal matrix is a reducing compartment

Peroxisomes are characterized by an abundance of hydrogen peroxide (H_2_O_2_)-producing oxidases [17]. In mammals, these oxidases participate in several metabolic pathways (*e.g.*, fatty acid β-oxidation, d-amino acid oxidation, and polyamine catabolism; [18]) and may produce massive amounts of H_2_O_2_, particularly in the liver [17]. Indeed, it has been estimated that, at least in some species, 20% of the O_2_ consumed by this organ may be channeled into these peroxisomal oxidases [17]. Hydrogen peroxide is a weakly reactive ROS but it can nevertheless modify proteins directly [19]. More importantly, H_2_O_2_ can undergo Fenton chemistry generating highly reactive radicals (*e.g.*, hydroxyl and carbonate anion radicals), which in turn can rapidly oxidize virtually any organic molecule [20,21].

To minimize oxidative damage, mammalian peroxisomes contain a small set of antioxidative enzymes (reviewed in Refs. [[22], [23], [24]]). One of these enzymes is catalase, one of the most abundant peroxisomal proteins, comprising 15% of the total protein mass of a liver peroxisome [25]. The other antioxidative enzymes found in mammalian peroxisomes are superoxide dismutase 1 (SOD1; [26]), soluble epoxide hydrolase 2 (sEH; [27]), peroxiredoxin 5 (PRDX5; [28]), and glutathione S-transferase κappa 1 (GSTk1; [29]).

Despite the relatively small repertoire of antioxidative enzymes present in mammalian peroxisomes and the large amounts of H_2_O_2_ produced in these organelles, cysteine residues in peroxisomal matrix proteins are maintained as reduced as those of cytosolic proteins, as first assessed using two cysteine-based redox probes, roGFP2 and Redoxfluor [30,31] and subsequentially verified in quantitative redox proteomics analyses [32]. Importantly, it was also shown that transient treatment of cultured cells with strong oxidants leads to reversible alterations in the redox state of peroxisomal matrix protein cysteine residues [30]. Clearly, there are mechanisms to repair oxidized protein cysteine residues in the peroxisomal matrix.

Cysteine residues of cytosolic, mitochondrial, and nucleoplasm proteins are maintained in a reduced state by two enzymatic systems [4,9,33]: 1) the thioredoxin reductase-thioredoxin system, and 2) the glutathione reductase-GSH-glutaredoxin system. Both systems use NADPH to reduce a thiol:disulfide oxidoreductase – a thioredoxin or a glutaredoxin – which in turn reduces oxidized protein cysteine residues (*e.g.*, protein disulfides and mixed disulfides with glutathione). Each reduction cycle results in the oxidation of the oxidoreductase which, therefore, must be reduced again by the upstream component of the system. In the thioredoxin system, the upstream component is thioredoxin reductase, whereas in the glutaredoxin system the reductant is GSH [4,9,33]. As discussed below, mammalian peroxisomes lack glutathione reductase and the same is probably true for thioredoxin reductase [34]. Thus, reducing intraperoxisomal proteins using a thioredoxin-based system would require either the free diffusion of a thioredoxin (a ∼10 kDa protein) across the peroxisomal membrane or a redox relay system to transfer reducing equivalents from cytosolic thioredoxin to some (unknown) luminal thiol:disulfide oxidoreductase, similarly to the situation in the bacterial Dsb disulfide bond formation system [35]. However, there are no data supporting the possibility that the peroxisomal membrane is freely permeable to small proteins, quite the contrary [36], and no peroxisomal transmembrane protein with the potential to act as a redox relay was ever found [[37], [38], [39], [40]].

In contrast to thioredoxins, glutaredoxins are efficiently reduced by GSH [3]. Therefore, a glutaredoxin or a glutaredoxin-like protein residing in the peroxisomal matrix could be reduced by cytosolic GSH, provided that the mammalian peroxisomal membrane is permeable to the GSH/GSSG pair. Evidently, this begs for two questions: 1) is there a glutaredoxin or a glutaredoxin-like protein in the mammalian peroxisomal matrix?; and 2) is the mammalian peroxisomal membrane permeable to GSH/GSSG? We will address these two questions later.

## 3 Glutathione-dependent enzymes in peroxisomes

The presence of glutathione-dependent enzymes in peroxisomes of several organisms is known for many years ([28,29,41]; see Table 1). Plant peroxisomes seem to have the largest number of this type of enzymes as assessed by proteomic analyses of subcellular fractions highly enriched in peroxisomes and *in vivo* subcellular localization experiments using fluorescently tagged proteins [[42], [43], [44], [45]]. Although additional work is still needed to confirm some of the available data (see Ref. [46] for a cautionary note on the *in vivo* subcellular localization experiments performed with the plant proteins), those studies found several glutathione S-transferases in *Arabidopsis thaliana* peroxisomes, namely members of the theta (GSTT1, GSTT2, and GSTT3), dehydroascorbate reductase (DHAR1), and lambda classes (GSTL2) (see Table 1). Members of the theta family are classical GSTs with both glutathione transferase and peroxidase activities towards hydrophobic electrophiles (*e.g.*, lipid hydroperoxides; [47]) whereas GSTL2 is a monomeric protein displaying glutaredoxin activity ([48,49]; see also [45]). The other GST member, DHAR1, uses GSH to reduce dehydroascorbate to ascorbate, one of the reactions of the glutathione-ascorbate cycle [50]. Interestingly, in addition to DHAR1, other enzymes of this cycle were also found in plant peroxisomes [44], including a small fraction of cytosolic NADPH-dependent glutathione reductase [51].

**Table 1:** Antioxidative enzymes found in peroxisomes from plants, fungi, and animals.

ENZYMES	PLANTS (*Arabidopsis thaliana*)	FUNGI (*Saccharomyces cerevisiae*)	ANIMALS (*Rattus norvegicus*)	REFERENCES
**GLUTATHIONE-DEPENDENT**				
	Glutathione *S*-transferase theta 1 (**GSTT1**) Glutathione *S*-transferase theta 2 (**GSTT2**) Glutathione *S*-transferase theta 3 (**GSTT3**) Glutathione S-transferase lambda 2 (**GSTL2**) Dehydroascorbate reductase 1 (**DHAR1**)	Glutathione *S*-transferase omega-like 1 (**GTO1**)	Glutathione S-transferase kappa 1 (**GSTk1**)	[29,38,42,45,47,57,121]
	Glutathione reductase (**GR**)	Glutathione reductase (**GLR1**)		[51,57]
**OTHER:**				
	Cu/Zn superoxide dismutase (**CSD3**)		Cu/Zn superoxide dismutase (**SOD1**)	[26,122,123]
	Catalase 1 (**CAT1**) Catalase 2 (**CAT2**) Catalase 3 (**CAT3**)	Catalase A (**CTA1**)	Catalase (**CAT**)	[121,[124], [125], [126], [127]]
	Epoxide hydrolase 3 (**EH3**)		Epoxide hydrolase 2 (**sEH**)	[121,128,129]
			Peroxiredoxin 5 (**PRDX5**)	[28]
	Ascorbate peroxidase isoform 3 (**APX3**) Ascorbate peroxidase 5 (**APX5**)			[130]
	Monodehydroascorbate reductase isoform 1 (**MDAR1**)			[131]
	Monodehydroascorbate reductase isoform 4 (**MDAR4**)			[131]

**Fig. 2:**
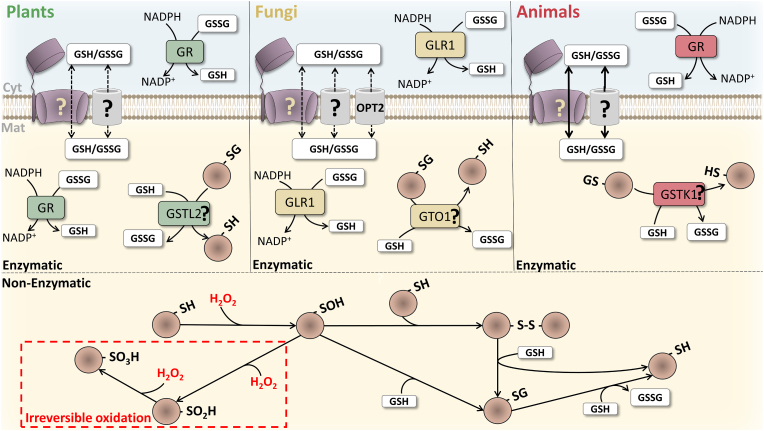
**– The protective role of glutathione against oxidative damage to protein thiol groups.** Glutathione-based enzymatic and non-enzymatic reactions with oxidized proteins are shown. With the exception of yeast OPT2, the identity of all the other peroxisomal glutathione pores/channels remains unknown (“?”). One of these pores might be the peroxisomal protein translocon (purple structures in the membrane), as hypothesized previously [9]. Regardless of their identities and mechanisms of transport (*i.e.*, pore/glutathione-specific transporter, unidirectional/bidirectional), the capacity of glutathione transporters in different organisms may be different, resulting in peroxisomal glutathione pools that may display different degrees of kinetic connectivity with the cytosolic pool. In rat liver, the permeability of the peroxisomal membrane to glutathione is relatively large, as assessed in *in vitro* experiments [36]. In yeast and plants, the kinetic connectivity of the two pools of glutathione may be low, at least under some physiological conditions, as suggested by the presence of glutathione reductase in their peroxisomes. Hydrogen peroxide oxidizes the thiol group of protein cysteine residues to the sulfenic (-SOH), sulfinic (-SO_2_H) and sulfonic (-SO_3_H) derivatives. There are no repair systems for protein sulfonates. Some protein sulfinates can be repaired by sulfiredoxin but this enzyme was never found in peroxisomes. Protein disulfides (-S-S- and -SG) can be reduced by GSH by both enzymatic and non-enzymatic mechanisms (see main text for details). Cyt – cytosol; GR and GRL1 – Glutathione reductase; GSH/GSSG – reduced/oxidized glutathione; GSTk1 – Glutathione S-transferase kappa 1; GSTL2 – Glutathione S-transferase lambda-2; GTO1 – Glutathione S-transferase omega-like 1; Mat – matrix; OPT2 – Oligopeptide transporter 2; SG – glutathionylated protein derivative; SH – reduced protein thiol group; SOH – sulfenic protein derivative; SO_2_H – sulfinic protein derivative; SO_3_H – sulfonic protein derivative; S–S – disulfide linked protein. (For interpretation of the references to color in this figure legend, the reader is referred to the Web version of this article.)

A simpler situation is found in yeasts (see Table 1 and Fig. 2). Peroxisomes from *Candida boidinii* contain PMP20, a peroxiredoxin that displays glutathione peroxidase activity [52] and, importantly, peroxisomes from this yeast were shown to contain GSH but no glutathione reductase [52]. Peroxisomes from *Saccharomyces cerevisiae* also contain GSH/GSSG, as assessed with a peroxisome-targeted sensor of the glutathione redox state [53]. Interestingly, however, the *S. cerevisiae* orthologue of *C. boidinii* PMP20, AHP1/TSA II/cTPx III (YLR109W) is probably not a peroxisomal protein [54,55]. Instead, *S. cerevisiae* peroxisomes contain a GST omega-like 1 protein (GTO1; [56,57]). The presence of GTO1 in *S. cerevisiae* peroxisomes is of interest because this atypical GST enzyme displays glutaredoxin-like activity ([58]; see also [59] and later). We note that *S. cerevisiae* peroxisomes were reported to contain also glutathione peroxidases 1 and 3 [60]. However, subcellular localization studies using GFP-fusion proteins do not corroborate this possibility [55,61]. Significantly, and similarly to the situation in plants*, S. cerevisiae* peroxisomes were recently shown to contain a small fraction of cytosolic NADPH-dependent glutathione reductase [57]. The presence of this enzyme in yeast (and plants; see above) peroxisomes might suggest that the organelle membrane is essentially impermeable to GSSG, similarly to the mitochondrial inner membrane. However, as discussed later, this may not be necessarily so.

The scenario with mammalian peroxisomes is the simplest one (see Fig. 2). Despite numerous biochemical/cell biology studies and proteomic analyses of highly pure liver and kidney peroxisomes performed over the last three decades, only one glutathione-dependent enzyme is presently known in mammalian peroxisomes [29,[37], [38], [39], [40]]. This is the above mentioned GSTk1. GSTk1 displays glutathione transferase and glutathione peroxidase activities in *in vitro* assays with a few substrates [29,62], a property that has been used to support the idea that GSTk1 may be an antioxidative enzyme [63,64]. In agreement with this, it has been shown that increasing the amount of GSTk1 in peroxisomes of mouse embryonic fibroblasts protects cells from ROS-induced cell death [65]. Very recently it was also proposed that GSTk1 may display glutathione:disulfide oxidoreductase activity ([66]; see later).

It should be noted that glutaredoxin-1 (GLRX1) (and thioredoxin (TXN)) were occasionally identified in the proteomic analyses of mammalian peroxisomes referred to above [[37], [38], [39]]. However, as trace amounts of cytosolic/mitochondrial/ER proteins are always found even in highly pure peroxisome preparations, it is possible that the GLRX1 (and TXN) protein detected in those proteomic analyses represents a cytosolic contamination. Importantly, no glutathione reductase was ever found in proteomic analyses of mammalian peroxisomes [[37], [38], [39], [40]] and accordingly, attempts to detect its activity biochemically in rodent peroxisomes yielded negative results [67]. Thus, any oxidized glutathione eventually produced in the mammalian peroxisomal lumen is probably not reduced *in situ*.

## 4 The mammalian peroxisomal membrane is promptly permeable to GSH/GSSG

Peroxisomes import and export a variety of metabolites and cofactors. They do so by 3 different mechanisms (reviewed in Refs. [68,69]). A few enzyme cofactors (*e.g.*, FAD and FMN) are probably co-imported together with the corresponding enzymes (note that peroxisomes can import already folded proteins; see Ref. [70] for a review). Some large/bulky metabolites (*e.g.*, ATP, CoA and acyl-CoAs) are transported by selective channels/transporters [68,69,[71], [72], [73], [74], [75], [76]]. Lastly, small metabolites (*e.g.*, isocitrate, glycerol-3-phosphate) are transported across the peroxisomal membrane by non-selective high-capacity pore-like transporters [68,69]. The exact molecular mass cut-off for these pore-like transporters may be somewhat different in different organisms [68,77]. Nevertheless, an accepted general rule for mammalian peroxisomes is that “solutes with molecular masses up to 300–400 Da” can diffuse freely across the organelle membrane [68] although, as discussed recently, the molecular shape and charge of a solute may also have some weight on this value [69].

Given the permeability characteristics of the mammalian peroxisomal membrane, it has been frequently assumed that GSH (307 Da) simply diffuses freely across the peroxisomal membrane via the non-selective, high-capacity pore-like transporters [24,34]. The fate of intraperoxisomal GSSG (613 Da) remained, for many years, more hypothetical. According to some authors, GSSG could either be reduced in peroxisomes (a hypothesis contingent on the presence of glutathione reductase in the peroxisomal matrix) or somehow exported back into the cytosol [24,34]. Recently, we used a biochemical strategy to address this problem [36]. Using a rat liver post-nuclear supernatant (PNS) containing the glutathione redox sensor PTS2-roGFP2-GLRX1 in both the cytosol and peroxisomes, we found that regardless of its localization, the sensor was rapidly reduced or oxidized when physiological amounts of GSH or GSSG, respectively, were added to the PNS. Furthermore, peroxisomal PTS2-roGFP2-GLRX1 that had been oxidized with GSSG was quickly reduced when the PNS was supplemented with both NADPH and glutathione reductase, thus suggesting that intraperoxisomal GSSG can exit peroxisomes to be reduced in the cytosol. These results suggest that the mammalian peroxisomal membrane is promptly permeable to both GSH and GSSG and, therefore, that the peroxisomal and cytosolic pools of GSH/GSSG are not kinetically insulated. Interestingly, side-by-side comparisons of the reduction/oxidation kinetics of the peroxisomal and cytosolic PTS2-roGFP2-GLRX1 pools revealed slight differences: whereas cytosolic PTS2-roGFP2-GLRX1 equilibrated with cytosolic GSH/GSSG almost instantly, peroxisomal PTS2-roGFP2-GLRX1 required 1–2 min to reach redox equilibrium. This finding explains why peroxisome-cytosol redox gradients can still be detected in metabolically active live cells [30,66], despite the large permeability of the mammalian peroxisomal membrane to GSH/GSSG.

The transporter(s) that allow GSH/GSSG to go across the mammalian peroxisomal membrane remain(s) totally enigmatic. Despite the scarcity of transmembrane proteins in peroxisomes (reviewed in Ref. [69]), mice or mammalian cells lacking one of these proteins or possessing a severe mutation in the corresponding gene do not display signs of a general peroxisome deficiency [69,[78], [79], [80], [81], [82], [83], [84], [85], [86]]. One possibility to explain these findings is that other peroxisomal antioxidant defenses (*e.g.*, catalase) compensate the lack of glutathione in the organelle (see Ref. [36]). It is also possible that there are two or more redundant glutathione transporters in the mammalian peroxisomal membrane and thus only the simultaneous knockout of the relevant genes will reveal a defect in glutathione transport across the peroxisomal membrane. Alternatively, and as proposed several years ago [9], glutathione might enter and exit the organelle (also) through the peroxisomal protein matrix translocon. Although we are still lacking structural information on this multisubunit transmembrane protein complex, the available biochemical data suggest that this translocon forms a large hydrophilic pore in the membrane through which newly synthesized (cytosolic) proteins reach the organelle lumen [87,88]. At first sight, this hypothesis may seem unlikely because all peroxisome-containing organisms possess such a translocon and, as explained above, the peroxisomal membranes of different organisms may display different permeabilities to glutathione. However, in the absence of information regarding the numbers of translocons *per* organelle in different organisms and the *in vivo* degree of saturation of the translocon by proteins *en route* to the peroxisome matrix, this hypothesis remains plausible.

Data on GSH/GSSG peroxisomal transporters in other organisms are likewise very limited. To the best of our knowledge, thus far, only one membrane protein was proposed to transport glutathione across the peroxisomal membrane. This is the *S. cerevisiae* OPT2 [53], a protein with no orthologue in mammals. Interestingly, it was proposed that OPT2 transports peroxisomal GSSG to the cytosol to be reduced, an idea that at the light of recent data suggesting that yeast peroxisomes contain glutathione reductase [57] might seem unlikely. However, recent work on the yeast *Debaryomyces hansenii* [89] indicates that, at least in one case, an intraperoxisomal regeneration system for a bulky metabolite can co-exist with a large-capacity membrane transporter for that same metabolite. Indeed, that yeast requires large amounts of NAD^+^ in the peroxisomal matrix for fatty acid β-oxidation and uses two strategies to achieve this requirement: 1) peroxisomal NADH is converted enzymatically to NAD^+^ *in situ*, yielding two small metabolites (malate and glycerol-3-phosphate) that are exported to the cytosol, and 2) peroxisomal NADH is simply exchanged by cytosolic NAD^+^. Thus, it is possible that peroxisomal GSSG in *S. cerevisiae* can also follow two pathways: 1) reduction *in situ* by peroxisomal glutathione reductase and 2) export to the cytosol, where it is reduced by cytosolic glutathione reductase.

## 5 How are reducing equivalents from GSH conveyed to oxidized protein cysteine residues in the peroxisomal matrix?

As explained above, cysteine residues in mammalian peroxisomal matrix proteins are maintained as reduced as those of cytosolic proteins. The same is true for *S. cerevisiae*, as assessed from a recent quantitative redox proteomics analysis [90], and measurements of the glutathione redox potential in yeast peroxisomes [53]. We are still lacking redox proteomics data for plant peroxisomes but as the thiol-disulfide redox potential in the matrix of these organelles is similar to that of the cytosol [[91], [92], [93]], an analogous situation may exist in plants. If there is glutathione in the peroxisomal matrix of all organisms, how are its reducing equivalents conveyed to oxidized protein cysteine residues? We are still missing many pieces of the glutathione-peroxisome puzzle to fully understand this aspect of peroxisome biochemistry. Nevertheless, it is probable that both enzymatic and non-enzymatic glutathione-based mechanisms play a role in this process, as discussed below.

### 5.1 Enzymatic glutathione-based mechanisms

Our knowledge on intraperoxisomal glutathione-based enzymes with the ability to transfer redox equivalents from glutathione to protein thiol groups remains very scarce and limited to the identification of some candidates. As stated above, *S. cerevisiae* peroxisomes contain GTO1, a monothiol omega-like GST [58]. The importance of GTO1 for yeast metabolism is highlighted by the fact that strains lacking this enzyme grow poorly on media containing oleic acid as an energy source, a condition in which large amounts of H_2_O_2_ are produced in the organelle matrix [56]. Yeast GTO1 displays glutaredoxin-like activity *in vitro* [58], raising the possibility that, similarly to human GST omega-1 [94], it can deglutathionylate protein substrates. Reduction of protein disulfide bonds might also be accomplished by yeast GTO1 either directly (see Ref. [95] for a mechanism of this type) or, more likely, indirectly, after a thiol-disulfide exchange reaction between the protein disulfide and GSH, as described for the reduction of mammalian ribonucleotide reductase by an engineered monothiol glutaredoxin 1 ([96]; reviewed in Ref. [3]).

A similar reasoning can be applied to *A. thaliana* GSTL2, an atypical monomeric GST that contains a catalytic cysteine residue [48,49]. GSTL2 also displays glutaredoxin-like activity *in vitro,* and thus, it might be part of the plant peroxisomal machinery that repairs oxidized protein cysteine residues. It is interesting to note that GrxC1, one of the ∼30 glutaredoxins present in *A. thaliana* [97], possesses a peroxisomal targeting signal type 1 [98,99], although, to the best of our knowledge, this protein was never found in proteomics analyses of plant peroxisomes.

In contrast to yeast GTO1 and plant GSTL2, mammalian GSTk1 lacks a cysteine residue at the active site. Thus, a classical glutaredoxin-like mechanism involving a direct nucleophilic attack of glutathionylated proteins by the enzyme is not possible. However, GSTs are versatile enzymes, and some can disrupt disulfide bonds by using an activated GSH molecule to perform the nucleophilic attack [3,100]. A recent study proposed that this is the case for GSTk1. Indeed, it was found that the presence of GSTk1 in peroxisomes increases the rate at which a peroxisome-targeted roGFP2 protein recovers its reduced state after a strong chemical oxidative insult leading the authors to conclude that GSTk1 possesses glutaredoxin-like activity [66]. Interestingly, however, in agreement with the absence of a peroxisomal phenotype in GSTk1-knockout mice [101], the basal peroxisomal thiol-disulfide redox potential in cells lacking GSTk1 is not altered [66], indicating that there are other mechanisms to keep cysteine residues of peroxisomal matrix proteins in the reduced state.

### 5.2 Non-enzymatic glutathione-based mechanisms

**Fig. 3:**
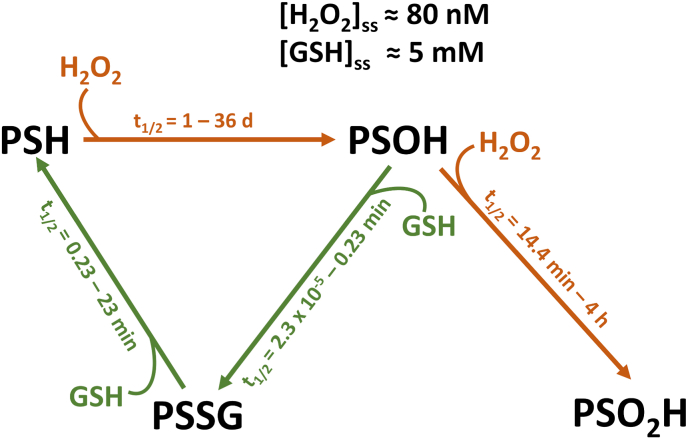
**– Non-enzymatic oxidation/reduction kinetics of protein thiol groups in peroxisomes.** The half-lives (t_1/2_) of the different species were calculated assuming peroxisomal steady-state (ss) concentrations of 80 nM and 5 mM for H_2_O_2_ and GSH [36], respectively, and the following second order rate constants: oxidation of cysteine (PSH) to sulfenic acid (PSOH) – 2.7 M^−1^ s^−1^ for non-catalytic cysteines [104] and 100 M^−1^ s^−1^ for active-site cysteine residues ([106]; see also [36]); oxidation of cysteine sulfenic (PSOH) to sulfinic acid (PSO_2_H) by H_2_O_2_ – 10^2^–10^4^ M^−1^ s^−1^ [106,107,132]; condensation of cysteine sulfenic acid (PSOH) with the thiol group of GSH – 6.7–10^5^ M^−1^ s^−1^ ([102,104]; see also [36]); and thiol-disulfide exchange – 0.1–10 M^−1^ s^−1^ [102]. Second-order rate constants were multiplied by the corresponding steady-state concentrations of GSH or H_2_O_2_ to obtain pseudo-first order constants (K), and these were converted to half-lives (t_1/2_ = ln2/K). Red and green colors indicate oxidation and reduction reactions, respectively. GSH – reduced glutathione; H_2_O_2_ – hydrogen peroxide; PSH – reduced protein; PSSG – glutathionylated protein; PSOH – protein sulfenic derivative; PSO_2_H – protein sulfinic derivative. (For interpretation of the references to color in this figure legend, the reader is referred to the Web version of this article.)

Although glutathione-based reactions are mostly catalyzed *in vivo*, glutathione can also participate in spontaneous/direct reactions such as condensation with cysteine sulfenic acids and thiol-disulfide exchange reactions [102]. Aiming at gauging the importance of these non-enzymatic reactions for intraperoxisomal protein redox homeostasis, we have recently developed a kinetic redox model of the mammalian peroxisome [36]. The results obtained suggest that indeed non-enzymatic mechanisms involving GSH are sufficient to protect intraperoxisomal protein cysteine residues from oxidation by H_2_O_2_. Even peroxisomal enzymes possessing catalytic cysteine residues, which are expected to be particularly sensitive to H_2_O_2_ (*e.g.*, the thiolases ACAA1 and SCPx and the bile acid-CoA:amino acid N-acyltransferase, BAAT) may be protected from H_2_O_2_-mediated oxidation by GSH in the absence of thiol:disulfide oxidoreductases. The reason for this counterintuitive conclusion is related to the relative physiological steady-state concentrations of GSH and H_2_O_2_ (∼5 mM versus 1–100 nM, respectively; [8,103]) and the rates at which these species react with the sulfenyl derivatives of cysteine residues, the first products of the reaction between a thiol group and H_2_O_2_ (see Fig. 3). GSH reacts with cysteine-sulfenic acid at rates of ∼10-10^5^ M^−1^ s^−1^ ([102,104]; see also [36]), whereas H_2_O_2_ oxidizes protein cysteine-sulfenic acid to cysteine-sulfinic acid (an irreversible cysteine oxidation product in peroxisomes due to the absence of sulfiredoxin in the organelle; [105]) at rates of about 10^2^-10^4^ M^−1^ s^−1^ [106,107]. Thus, even if we take the smallest rate constant for the reaction between GSH and cysteine-sulfenic acid (*i.e.*, 10 M^−1^ s^−1^) and the largest one for the reaction between H_2_O_2_ and cysteine-sulfenic acid (*i.e.*, 10^4^ M^−1^ s^−1^), the fact that GSH is 50 000 to 5 000 000-fold more abundant than H_2_O_2_ means that most protein sulfenic acids will react preferentially with GSH (yielding a glutathionylated protein) instead of being further oxidized to protein sulfinic acids. Glutathionylated proteins can then be completely reduced by direct reaction with GSH. Although spontaneous thiol-disulfide exchange reactions are relatively slow when compared with those catalyzed by thiol:disulfide oxidoreductases such as glutaredoxin (0.1–10 M^−1^ s^−1^ and 10^4^-10^6^ M^−1^ s^−1^, respectively; [102]), the large physiological concentration of GSH (∼5 mM; [8]) results in a pseudo-first order rate constant for the non-enzymatic reduction of a glutathionylated protein by GSH of 5x10^−4^-5x10^−2^ s^−1^, which corresponds to a half-life of just 0.23–23 min.

## 6 Glutathione is more important than catalase in protecting peroxisomal protein cysteine residues from H_2_O_2_-induced oxidation

Unexpectedly, the kinetic simulations referred to above also revealed that the non-enzyme assisted action of glutathione inside the mammalian peroxisome provides more protection against H_2_O_2_ than catalase itself [36]. Indeed, although the absence of catalase in peroxisomes increased the intraperoxisomal H_2_O_2_ concentration from ∼80 nM to ∼1 μM, which led to the massive oxidation of peroxisomal matrix proteins, having a peroxisomal membrane permeable to cytosolic glutathione was sufficient to protect intraperoxisomal proteins from H_2_O_2_-induced oxidation even in the absence of a glutaredoxin. This does not mean that catalase has a redundant/non-important function inside peroxisomes. By maintaining the intraperoxisomal concentration of H_2_O_2_ at low levels, catalase decreases the fraction of H_2_O_2_ that can potentially undergo Fenton chemistry. Also, by scavenging most of the H_2_O_2_ generated by peroxisomal oxidases, catalase decreases the amount of peroxisomal H_2_O_2_ that reaches the cytosol by ∼94% [36], thus sparing NADPH consumption by the cytosolic H_2_O_2_-scavenging peroxiredoxin and glutathione peroxidase systems. The amount of NADPH saved by peroxisomal catalase may be quite substantial. For instance, if we extrapolate biochemical data from rat liver (380 nmol of peroxisomal H_2_O_2_ produced *per* gram of liver *per* min; [108]) to adult humans (liver weight ∼1.5 Kg) and consider that the human liver resting metabolic rate is ∼1/4 that of rat liver [109] we conclude that 0.2 mol of H_2_O_2_ are produced every day in human liver peroxisomes. This is equivalent to 230 mL of the 3% (w/v) H_2_O_2_ solution that can be acquired in pharmacies. In the absence of peroxisomal catalase, all this H_2_O_2_ would reach the cytosol where its neutralization would cost 0.2 mol of NADPH or about 3 g of glucose/day, assuming that all the NADPH derives from the pentose phosphate pathway (1 mol of glucose (180 g) → 12 mol of NADPH). In the presence of catalase only 180 mg of glucose/day are consumed by the cytosolic NADPH-dependent reductive systems.

## 7 Conclusions and future perspectives

Here, we summarize data on the peroxisome-glutathione relationship. Although our focus was the mammalian peroxisome, data from other organisms were also analyzed. A major conclusion that can be drawn from these comparisons is that data gathered on the glutathione-peroxisome topic in a given organism cannot be easily extrapolated to other organisms. It is long known that peroxisomes are some of the most plastic organelles of the eukaryotic kingdom that can hold widely different enzyme repertoires in different organisms [110,111]. However, some basic functions (*e.g.*, β-oxidation of fatty acids, presence of multiple oxidases and catalase) were mostly conserved throughout evolution [111], and thus the realization that important aspects of glutathione biochemistry in a given organism, are not conserved in others is somewhat unexpected.

Despite all the differences in the glutathione-based enzyme repertoire and, possibly, also in the permeabilities of the peroxisomal membrane to GSH/GSSG, it is evident that all organisms characterized up to now have glutathione in their peroxisomes. This, *per se*, is important because the available data suggest that the mere presence of glutathione within the organelle is already sufficient to protect peroxisomal proteins from oxidation by H_2_O_2_. The protective role of glutathione also offers a straightforward explanation for the fact that humans with acatalasia exhibit essentially no peroxisome-related phenotypes [112], a property that is also true for animal models such as catalase-knockout mice [113]. In principle, the absence of catalase in peroxisomes leads to a massive increase in intraperoxisomal H_2_O_2_, which, in turn, should trigger extensive oxidation/inactivation of peroxisomal enzymes [36]. However, no such oxidation/inactivation occurs due to the robust protective effect of glutathione (see Fig. 3; [36]).

In addition to H_2_O_2_, other ROS are certainly generated in the peroxisomal matrix or reach the organelle from the cytosol, and these ROS should also be eliminated. Whereas in some cases there are no reasons to believe that glutathione is involved in these defense mechanisms, in others this still needs to be investigated. For reactive species such as superoxide anion and some epoxides, mammalian peroxisomes are equipped with SOD1 and epoxide hydrolase 2 which convert those species into H_2_O_2_ and the corresponding vicinal diols, respectively [26,114]. Superoxide anion can also react with glutathione but, in the absence of SOD1, it is unlikely that glutathione plays any protective role against this ROS [115]. Epoxides, however, can also be rapidly conjugated to GSH by several GSTs [3,116] and thus, it would be important to determine whether GSTk1 also displays such an activity. Reactive species like hydroperoxides and peroxynitrite can, in principle, be efficiently neutralized by peroxisomal PRDX5 [[117], [118], [119]]. However, peroxiredoxins such as PRDX5 are generally supported by the thioredoxin system which reduces the oxidized peroxiredoxin at each catalytic cycle [120]. As stated above, no thioredoxin reductase was ever found in mammalian peroxisomes and thus exactly how PRDX5 functions in peroxisomes remains unknown. One possibility to explain this enigma is that oxidized PRDX5 is reduced by GSH [34] but experimental data to support this hypothesis are still lacking.

Many other questions remain on the peroxisome-glutathione topic. An obvious one regards the identity of the transporters that allow GSH/GSSG to go across the peroxisomal membrane. It is expected that a systematic knockout of genes encoding mammalian peroxisomal membrane proteins will soon lead to the identification of such (a) protein(s) [80], although problems may be encountered if the long-sought glutathione transporter is the protein translocon through which newly synthesized proteins are imported into the organelle [9]. Another important issue that remains unclear regards the enzymatic machinery that conveys redox equivalents from intraperoxisomal GSH to protein cysteine residues. Although it seems clear that the simple presence of glutathione in the peroxisomal matrix already provides an efficient protection against H_2_O_2_-induced oxidation, other more aggressive ROS may require more robust enzyme-based defenses. Yeast GTO1 and plant GSTL2 are, at present, the best candidates to catalyze glutathione-based repair of oxidized proteins, but experimental data to support this possibility are still lacking. In mammals, it was recently proposed that GSTk1 plays such a role but, likewise, the mechanism used by GSTk1 to maintain protein cysteine residues in the reduced state remains unknown. These and other aspects must be clarified if we are to fully understand the redox homeostasis mechanisms that operate inside the peroxisome.

## Authorship

MJF – conceptualization, investigation, writing - original draft, review & editing.

TAR, AGP, ARS, BGV – investigation, writing - review & editing.

TF – conceptualization, investigation, writing - original draft, review & editing.

JEA – conceptualization, investigation, writing - original draft, review & editing, funding acquisition.

All authors have contributed to the manuscript and approved the submitted version.

## Declaration of competing interest

The authors declare that they have no known competing financial interests or personal relationships that could have appeared to influence the work reported in this paper.

## Data Availability

No data was used for the research described in the article.
